# Healthcare utilisation, follow‐up of guidelines and practice variation on rhinosinusitis in adults: A healthcare reimbursement claims study in The Netherlands

**DOI:** 10.1111/coa.13453

**Published:** 2020-01-20

**Authors:** Nina M. Kaper, Mark C. J. Aarts, Robert J. Stokroos, Geert J. M. G. van der Heijden

**Affiliations:** ^1^ Department of Otorhinolaryngology and Head & Neck Surgery University Medical Center Utrecht Utrecht The Netherlands; ^2^ Department of Otorhinolaryngology Jeroen Bosch Hospital ‘s Hertogenbosch The Netherlands; ^3^ Department of Social Dentistry Academic Center for Dentistry Amsterdam University of Amsterdam and VU University Amsterdam The Netherlands

**Keywords:** clinical practice patterns, clinical practice variation, costs, epidemiology, evidence‐based medicine, evidence‐based practice, health expenditure, health insurance reimbursement, healthcare utilisation, insurance claim reporting, practice guideline, prevalence, sinusitis, volumes

## Abstract

**Objectives:**

To provide insight into healthcare utilisation of rhinosinusitis, compare data with clinical practice guideline recommendations and assess practice variation.

**Design:**

Anonymised data from claims reimbursement registries of healthcare insurers were analysed, from 1 January 2016 until 31 December 2016.

**Setting:**

Secondary and tertiary care in the Netherlands.

**Participants:**

Patients ≥18 years with diagnostic code “sinusitis.”

**Main outcome measures:**

Healthcare utilisation (prevalence, co‐morbidity, diagnostic testing, surgery), costs, comparison with guideline recommendation, practice variation.

**Results:**

We identified 56 825 patients, prevalence was 0.4%. Costs were € 45 979 554—that is 0.2% of total hospital‐related care costs (€21 831.3 × 10^6^). Most patients were <75 years, with a slight female preponderance. 29% had comorbidities (usually COPD/asthma). 9% underwent skin prick testing, 61% nasal endoscopy, 2% X‐ray and 51% CT. Surgery rate was 16%, mostly in daycare. Nearly, all surgical procedures were performed endonasally and concerned the maxillary and/or ethmoid sinus. Seven recommendations (25%) could be (partially) compared to the distribution of claims data. Except for endoscopy, healthcare utilisation patterns were in line with guideline recommendations. We compared results for three geographical regions and found generally corresponding rates of diagnostic testing and surgery.

**Conclusion:**

Prevalence was lower than reported previously. Within the boundaries of guideline recommendations, we encountered acceptable variation in healthcare utilisation in Dutch hospitals. Health reimbursement claims data can provide insight into healthcare utilisation, but they do not allow evaluation of the quality and outcomes of care, and therefore, results should be interpreted with caution.

**Key points**

- We assessed healthcare utilisation for rhinosinusitis in the Netherlands, based on reimbursement claims registries for healthcare insurers.
- We obtained data on >99% of healthcare providers for 2016.
- Based on our data, there seems to be no structural deviation from most important clinical practice guideline recommendations.
- We found limited and acceptable practice variation.
- Healthcare reimbursement claims contain insufficient information to determine healthcare quality and treatment outcomes.

## 1 INTRODUCTION

Rhinosinusitis is defined as symptomatic inflammation of the nasal cavity and paranasal sinuses. Acute rhinosinusitis (ARS) lasts <4 weeks, chronic rhinosinusitis (CRS) >12 weeks, patients with four or more episodes of ARS per year without symptoms in between are classified as recurrent acute rhinosinusitis (RARS). Patients with a prolonged or complicated course (eg meningitis, brain abscess, orbital cellulitis and orbital abscess) can also be distinguished.^1^, ^2^ Two or more symptoms should be present, one of which should be either rhinorrhea in ARS and RARS (anterior/posterior or both) or nasal blockage in CRS. Facial pain‐pressure‐fullness or loss of smell can also be present. For ARS and RARS, the diagnosis is confirmed by symptoms, for CRS by signs of inflammation at anterior/posterior rhinoscopy and/or pathological findings on CT.^1^, ^2^ There is a distinction between CRS with nasal polyps and without nasal polyps.^1^, ^2^ Worldwide, RS is a common disease, with a reported incidence of around 12%.^1^ Of RS, ARS is most common and patients usually present themselves in primary care, at their general practitioner.^1^ For CRS, a prevalence of 2% (defined with ICD‐9 codes for primary care and referral centres) to 11% (defined by self‐reporting) is reported, although there is a deficit in studies describing the prevalence of CRS in European countries.^2^, ^3^, ^4^ RARS is less common, with a reported incidence of 0.03%.^5^

Multiple clinical practice guidelines (CPGs) have been developed to guide and support clinical practice for RS, reduce practice variation and ultimately lead to better treatment outcomes.^6^ In the Netherlands, a national CPG is available (CBO 2010), providing recommendations only on CRS.^7^ Previous research shows that this CPG is used by most Dutch otolaryngologists.^8^, ^9^ In 2010, 61% of them reported being familiar with the CBO CPG.^8^ More recently, research showed that 96% of Dutch otolaryngologists are aware of this CPG, with sufficient to good adherence to its recommendations.^9^ However, data concerning actual CPG compliance in daily practice are lacking.^9^ Despite CPGs that drive healthcare utilisation patterns, local or regional practice variations may exist, or systematic deviation from the CPG may occur.

In this study, we will use data from healthcare reimbursement claims registries of Dutch healthcare insurers to provide insight into the volume and cost of the RS‐related healthcare utilisation in Dutch hospitals. We will compare results between different hospital types and regions to detect practice variation. In a previous study, healthcare reimbursement claims data have been used to assess non‐adherence to guideline recommendations; therefore, we will compare our data to Dutch recommendations from the CBO 20120 guideline on CRS to detect potential deviations from protocol.^7^, ^10^

## 2 METHODS

### 2.1 Ethical considerations

Under Dutch Law for Medical Research with Humans, it is allowed to process personal data for statistical and scientific analysis, provided data are not traceable back to individuals.^11^ Data were provided, processed and analysed by Vektis, which is the national business intelligence centre of the Dutch healthcare insurers.^12^ Data safety and security were guaranteed by Vektis. As we had no access to individual patient data, patient anonymity is guaranteed.

### 2.2 Utilisation of health care for rhinosinusitis

#### 2.2.1 Data extraction

We obtained data from Vektis, which collects and analyses healthcare reimbursement claims from almost all Dutch healthcare insurers, with coverage of >99% of healthcare providers.^12^ The reimbursement procedure for healthcare insurance is the same across the Netherlands. Medical conditions, including RS, are invoiced as diagnostic codes, based on ICD‐10 codes.^13^, ^14^ Healthcare providers invoice all activities linked to this diagnostic code (eg diagnostic procedures, surgical interventions) to the insurer of the patient. Under Dutch Law, basic health insurance is legally required for all citizens and all RS‐related health care is covered by this insurance.

In September 2018, we obtained data for the year 2016 (1 January 2016 to 31 December 2016), for patients ≥18 years (determined on 30 June 2016) from secondary and tertiary healthcare. We received data on all reimbursement claims for the diagnostic code “sinusitis,” filed until 31 May 2018. The reimbursement claims coding system does not distinguish between different subtypes of RS, so ARS, RARS, CRS and complicated RS are all covered by this code.

Data on age, gender and co‐morbidity were obtained. To identify patients with comorbidities, we used a nationwide registration system on the use of pharmaceuticals (FKG).^15^ Insured persons with a chronic condition were identified based on reimbursement claims of certain medication that is known to be used in a chronic condition. We extracted data on chronic obstructive pulmonary disease (COPD), asthma, diabetes mellitus and cardiac conditions since these are comorbidities known to influence decisions on surgical treatment strategy.

We obtained data on nasal endoscopy, allergy testing (skin prick), radiographic imaging (CT and X‐ray), the number and type of surgical procedures and related hospital admissions.

#### 2.2.2 Comparison with Dutch CPG

We extracted 28 recommendations on the diagnosis and treatment of CRS for adults (see Supporting Information S1) and compared these to our data.

#### 2.2.3 Practice variation and comparison between hospitals

**Figure 1:**
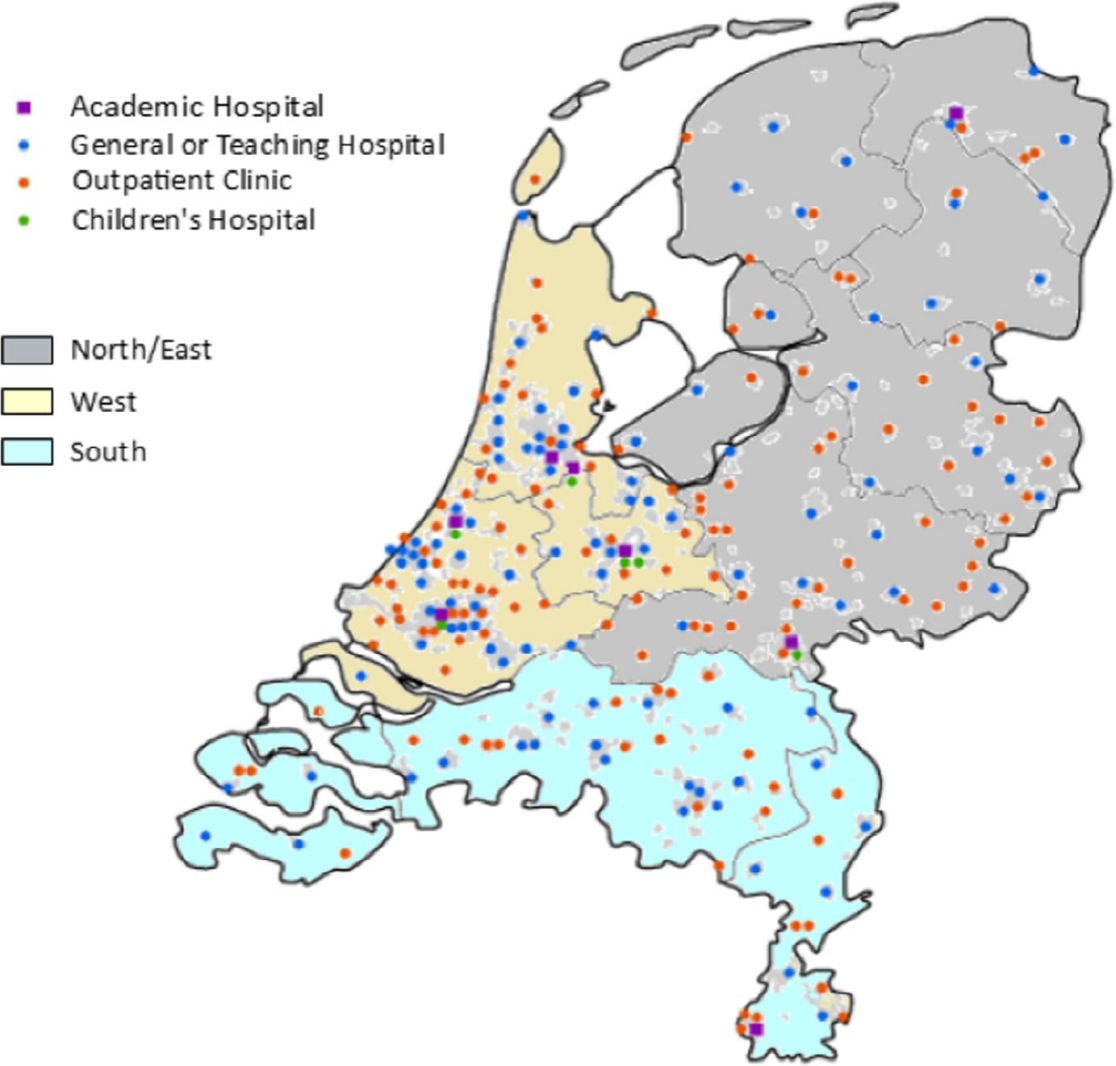
Hospitals and regions in the Netherlands

We compared practice patterns between different hospital types in the Netherlands and between three regions; South, North/East and West. (see Table 1).^16^ North/East and South both have large rural areas, whereas West is more urbanised and densely populated. (see Figure 1).

**Table 1:** Characteristics of patients with rhinosinusitis in 2016

	Hospitals	Patients	Prevalence (%)^a^	Male n (%)	Age (mean, SD)	Co‐morbidity n (%)^b^	Costs (%)^c^	Costs^d^
Hospital type								
General	46	24 781	NP	12 019 (49)	52 (16)	7175 (29)	190 (41)	768
Teaching	25	25 318	NP	12 180 (48)	52 (16)	7282 (29)	204 (44)	805
Academic	8	4 376	NP	2260 (52)	51 (16)	1728 (39)	46 (10)	1048
Private	8	3052	NP	1371 (45)	51 (15)	676 (22)	20 (4)	654
Region								
West	NP	24 806	0.39	NP	52 (16)	6996 (28)	191 (42)	769
South	NP	14 814	0.50	NP	52 (16)	4249 (29)	121 (26)	817
North/East	NP	17 465	0.41	NP	52 (16)	5478 (31)	148 (32)	847
Total								
	87	56 852^e^	0.42	27.502 (48)	52 (16)	16 643 (29) −13 026 (23)^f^ −3617 (6)^g^	460	809

Note

n: number of patients. %: percentage of total.

Abbreviations: NP, not provided; SD, standard deviation.

a Total population North/East: 4 209 597, West 6 435 258, South 2 940 218 (Source: CBS Statline^16^).

b Patients with either COPD and/or asthma or diabetes and/or cardiac conditions, or both.

c Total costs in million euro's.

d Average cost per patient in euro's.

e Total patients is lower than the sum of the above data, since 103 patients(<1%) visited multiple hospitals and 233 patients (<1%) were treated in multiple regions.

f COPD and/or asthma.

g Diabetes and/or cardiac conditions.

#### 2.2.4 Data analysis

Performed in microsoft excel 2010.^17^ Due to the large number of patients, 95% and even 99% confidence intervals are narrow, which results in differences of 1% already being statistically significant.^18^ Since this is principally a descriptive study of the volume and the costs of care for RS in Dutch Hospitals, we will neither go beyond presenting the data distributions nor provide data on test statistics from statistical analyses.

## 3 RESULTS

We found a total of 56 852 patients with RS, that is a prevalence of 0.4% (total population ≥18 years of 13 585 073).^19^ This accounts for 8% of patients that visited an otolaryngologist in the year 2016.^20^ Costs of RS were €45 979 554—which is 0.2% of total hospital‐related health care (€21 831.3 × 10^6^).^21^ Characteristics of patients can be found in Table 1. There was a slight female preponderance. Most patients were below 75 years old. Patients without co‐morbidity were substantially younger than patients with co‐morbidity (mean age, respectively, 49 vs 61 years of age). COPD and/or asthma was more common than diabetes and/or cardiac conditions.

On average, patients visited the outpatient clinic 1, 3 times. 84% of patients visited the outpatient clinic more than one time, while 44% had three or more visits. For results of diagnostic testing, see Table 2. Details of surgical vs non‐surgical patients can be found in Table 3. Surgery claims can be found in Table 4. Surgery was usually limited to the maxillary and ethmoid sinus; the frontal and sphenoid sinus were rarely operated. External sinus surgery was performed in <1% of cases. It was not possible to differentiate between solitary procedures, combined procedures (eg maxillary sinus and ethmoid surgery) and revision surgery. The majority of procedures were performed within daycare (see Table 5).

**Table 2:** Diagnostic testing for all patients with rhinosinusitis in 2016 (n = 56 852^a^)

Diagnostic test	N (%)
Skin prick test	5336 (9)^b^
Nasal endoscopy (1 or more)	34 659 (61)
X‐ray	1201 (2)
CT scan	29 148 (51)
CT scan (twice or more)	1704 (3)
Endoscopy + CT	17 866 (31)

Note

N: number of patients. %: percentage of total.

Abbreviation: CT, computed tomography.

a Sum of patients with diagnostic testing is higher, since 103 patients (<1%) visited multiple hospitals.

b Mean age 43 y.

**Table 3:** Surgical vs non‐surgical care for all patients with rhinosinusitis seen in 2016 (n = 56 852^a^)

	Surgery (N = 9396)	No surgery (N = 47 564)
Age (mean [SD])	50 (16)	52 (26)
Co‐morbidity^b^ N (%)	2577 (27)	14 097 (30)

Note

n: number of patients. %: percentage of total.

Abbreviations: SD, standard deviation.

a Sum of operated/non‐operated patients is higher, since 103 patients (<1%) visited multiple hospitals.

b COPD and/or asthma, diabetes and/or cardiac conditions.

**Table 4:** Surgical procedures (multiple interventions per patient)

Surgical procedure	Claims (%)
Endonasal maxillary and/or ethmoid	14 300 (82.0)
Polyp extraction	1484 (8.0)
Endonasal (or radical) frontal, or sphenoid sinus^a^	982 (5.5)^b^
Antral lavage	588 (3.0)
External frontal or ethmoid sinus	97 (0.6)^b^
Radical maxillary sinus^c^	44 (0.3)
Total	17 495
Per patient	18
Range	1‐21

Note

%, percentage.

a For example Halle, Mosher, Vacher.

b < 1% of data missing.

c Caldwell‐Luc.

**Table 5:** Peri‐operative care (n = 9396)

	N (%)
Hospital admission	
Daycare	6125 (65)
Hospital stay	
1 night	155 (2)^a^
2 nights	2806 (30)
3 nights or more	332 (4)

Note

n: number of operated patients. %: percentage.

a <1% missing data.

### 3.1 Comparison with Dutch CRS guideline

**Figure 2:**
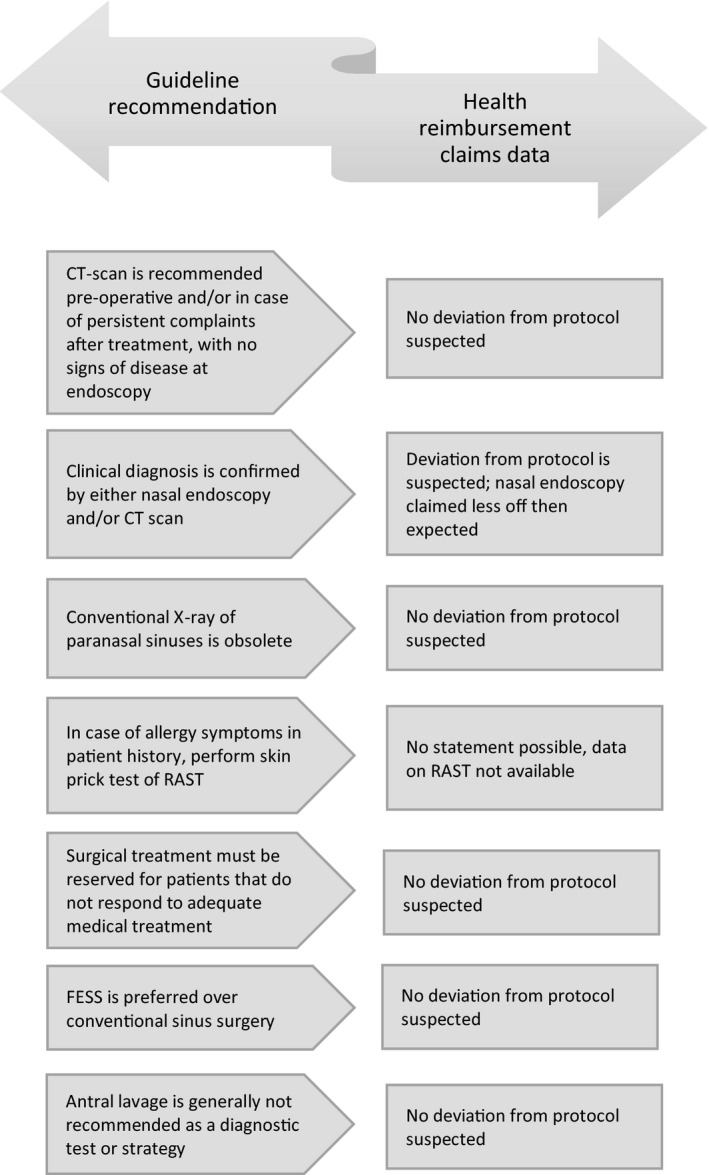
Guideline recommendations compared to health reimbursement claims

Seven recommendations (25%) could be (partially) compared to the distribution of claims data^7^. See Figure 2.

### 3.2 Healthcare utilisation compared by hospital and region

Patient characteristics can be found in Table 1. Most patients were treated in general/teaching hospitals and in the Western region, which reflects population density and hospital distribution in the Netherlands.(see Figure 1) Prevalence was similar across regions, with comparable patient population (based on age and co‐morbidity), while costs per patient were lower in the denser Western region.

### 3.3 Diagnostic testing compared by hospital and region

We found no important differences in claims for allergy testing by hospital type or region.

Nasal endoscopy was claimed most often in academic hospitals (75%) and private clinics (65%), and the least in general/teaching hospitals (60%). Nasal endoscopy was claimed somewhat more frequently in North/East and South (65% and 61%) compared to West (57%). CT scanning was claimed least in academic hospitals (27%) and was similar for other hospital types (49%). Claims for CT scans slightly varied across regions: North/East 44%, West 49% and South 52%. (also see Supporting Information S1).

### 3.4 Surgical procedures compared by hospital and region

Surgical procedures were claimed least by private clinics (12%) and more often in other hospital types (teaching 17%, general 16%, academic 14%). Co‐morbidity was present in 37% of operated patients in academic hospitals, vs 20% in private clinics. Surgery rates varied slightly between regions (West 15%, North/East 17% and South 18%), while co‐morbidity of operated patients did not differ. In academic hospitals, relatively many external, sphenoid and frontal sinus surgeries were performed (24%, vs 0.5%‐6% in other hospitals) and antral lavage was performed more often (7% vs 1%‐3% in other hospitals). There were no differences in type of surgical claims between regions. Except for academic hospitals, the majority of surgeries was performed within daycare. In the Western region, 77% of surgery was performed within daycare, whereas in the North/East and South this was, respectively, 53% and 61%.

## 4 DISCUSSION

### 4.1 Synopsis of key findings

We set out to assess healthcare utilisation and costs of RS for patients ≥18 years in secondary and tertiary care, based on healthcare reimbursement claims data, with a coverage of >99% of all healthcare providers. We discovered a lower prevalence than expected from previous studies.^1^, ^2^, ^3^, ^4^ Our study population was overall relatively young and healthy, which is comparable to previous studies.^1^, ^2^, ^3^, ^4^ Costs were less than one per cent of all Dutch hospital‐related health care. For 25% of the recommendations in the Dutch CPG on CRS, diagnostic and treatment patterns could be (partially) compared using these Data.^7^ Except for endoscopy, healthcare utilisation patterns showed no structural deviation from CPG recommendations, which is corroborated by limited regional practice variation.^7^ However, our study shows major limitations, and on top of that, reimbursement claims are based on financial parameters and therefore do not allow evaluation of the quality and outcomes of health care.

### 4.2 Comparison with other studies

Previous studies reported a higher prevalence of RS, although with the use of different methods. Less stringent definitions were used, studies were performed in primary care, or relied on self‐reported symptoms.^1^, ^2^, ^3^, ^4^ Age and gender distribution corresponded to previous studies.^2^, ^22^, ^23^ Allergy testing was encountered less than expected based on literature, but data on RAST are missing and allergy testing is probably invoiced using the diagnostic code “allergic rhinitis” which was not included in our study.^2^, ^7^, ^13^, ^14^

Results on nasal endoscopy are consistent with a study performed in the United States (concerning community and academic practice).^24^ Nasal endoscopy is registered by Dutch otolaryngologists themselves which might lead to under‐registration due to limited time and lack of financial incentive. However, this only partially explains the low number of registered endoscopies.

Surgery rate was comparable to a study in the United States.^23^ Previous studies showed much higher surgery rate variation, that is, in the United States up to three times, in Finland up to four times and in Canada up to two times higher.^25^, ^26^, ^27^

### 4.3 Variation by hospital type and region

In the Netherlands, most patients visit general and teaching hospitals; private clinics are not very common. There are eight academic hospitals in the country; these have an important function as referral centres for other hospital types (tertiary care), and therefore perform more complex care, which is reflected by our results.

We found little variation in the geographical prevalence of RS, which was to be expected since the geographic area of the Netherlands is small. For diagnostic testing, we found acceptable differences ranging from 1% to 8%. For surgery, rate differences were even smaller, being 2% or less. We did find a remarkable variation in the number of patients treated within daycare, which can be explained by the fact that in some regions patients generally live further away from the hospital.

### 4.4 Strength and Limitations

We had access to a large database that covered more than 99% of patients in the Netherland; therefore, we can present an almost complete overview of all RS‐related care. Our study is the first to assess healthcare reimbursement claims data for RS in the Netherlands. However, major limitations have to be addressed.

First, the data are derived from reimbursement claims, which are financial outcome measures. In addition, incorrect registration might have occurred; therefore, the data show an approximation of the actually delivered care. Besides, the data represent health care at a population level and are too limited to assess treatment patterns our outcomes.

Second, we compared our data to recommendations from a CPG on CRS, while we included patients in secondary and tertiary care based on the diagnostic code “sinusitis.” This code encompasses patients with ARS, RARS, CRS, patients with a duration of complaints between 4 and 12 weeks and patients with complicated RS. Due to the healthcare structure in the Netherlands, we can argue that the majority of patients in Dutch otolaryngology practice probably suffer from prolonged RS or CRS. Patients with complaints of RS must always first present to their GP and only after referral they may visit an otolaryngologist. According to their CPG, GPs only refer patients with three or four episodes of ARS, patients with a suspected complicated course of disease and patients with duration of complaints of 8 weeks or more.^28^ Therefore, patients with ARS will rarely be referred unless RARS or a complication is suspected, which is known from previous literature to be very rare.^1^, ^2^, ^5^ Consequently, we felt that the recommendations of the CPG on CRS could be compared to our claims data.

Third, we might have missed patients with RS that were registered under a different diagnostic code, for example “allergic rhinitis”.^13^, ^14^

Fourth, due to our cross‐sectional design, it was not possible to track the course of disease for individual patients, so patients visiting the hospital in 2016 might have undergone diagnostic testing or surgery in 2015 or 2017. Therefore, an underestimation of diagnostic testing and surgery cannot be precluded.

Fifth, since the Dutch healthcare structure varies from those in other countries, our results might not be extrapolated to other countries.

Sixth, since adherence to the Dutch CPG is evaluated in the context of a 5‐year quality assessment of Dutch otolaryngologists, this might have influenced our results and explain the limited practice variation.

Finally, our research neither evaluates whether the care invoiced was actually provided, nor whether it was needed. To assess what health care was delivered to the patients and whether diagnostic tests and interventions were indicated according to CPG recommendations, extensive chart review by field specialists would have to be performed.

### 4.5 Implications

The general public and especially patients visiting an otolaryngologist can benefit from the new insights of this study and be reassured by the fact that we found little structural deviation from CPG recommendations.

Otolaryngologists can use our results for a better understanding of RS‐related health care and comorbid diseases. Also, it shows in what way reimbursement claims can be used to assess health care. In the future, they should be aware that secondary use of reimbursement claims data might increase and therefore adequate registration remains important.

Outcomes of this study can help CPG authors and board members in designing new or improved methods for healthcare delivery and registration in RS, from which patients eventually will benefit. For example, further embedding guideline adherence as an evaluation tool in quality assessments might increase the adoption of evidence‐based CPGs. Methods to assign these quality benchmarks have been recently developed.^29^

For healthcare insurers and policymakers, it is important to realise that healthcare reimbursement claims data are too limited to assess quality of care or evaluate treatment outcomes. Our results also indicate the effects of market forces used to decrease healthcare costs. In line with competition between hospitals is higher in the Western region, we found decreased costs per patient, further contributing to a decrease of the total healthcare budget. Also, the low prevalence of RS in secondary/tertiary care, compared to the higher prevalence in previous studies (situated at population level or in primary care), implies that most patients with RS are treated by their GP and not by an otolaryngologist.^1^, ^2^, ^3^, ^4^ This demonstrates a high level of cost‐effectiveness of the Dutch healthcare system.

For researchers, these results add to the existing knowledge about RS and can be used as a foundation for formulating research priorities. Our study can also serve as an example for future studies on healthcare reimbursement claims.

## CONFLICT OF INTEREST

None to declare.

## AUTHOR CONTRIBUTIONS

NK and GH involved in concept of study design; NK involved in data acquisition; NK, MA, GH and RS analysed/interpreted the data; NK drafted the manuscript; NK, MA, GH and RS involved in critical revision of the manuscript; NK, MA, GH, RS involved in final approval.

## Data Availability

The data supporting the findings of this study are available from the corresponding author upon reasonable request.
