# Supplementary material for: Healthcare utilisation, follow‐up of guidelines and practice variation on rhinosinusitis in adults: A healthcare reimbursement claims study in The Netherlands
Source: Clin Otolaryngol. 2020 Jan 20;45(2):159–66. doi: 10.1111/coa.13453 (PMC9328289; doi:10.1111/coa.13453)
Supplement: Supplementary file 1 [file COA-45-159-s001.docx]

**Supplementary material**

1. Results for hospital type and region.

|  | | ***Age groups (years)*** | | | | | ***Comorbidity*** | ***Diagnostic testing†*** | | | | | | | | | ***Outpatient clinic visits†*** | | | | | | ***No surgery*** | | | ***Surgery*** | | | | ***Admission days (surgery)†*** | | | | | ***Intervention pp*** |
| --- | --- | --- | --- | --- | --- | --- | --- | --- | --- | --- | --- | --- | --- | --- | --- | --- | --- | --- | --- | --- | --- | --- | --- | --- | --- | --- | --- | --- | --- | --- | --- | --- | --- | --- | --- |
|  |  | ***18-30*** | ***31-45*** | ***46-60*** | ***61-75*** | ***>75*** | ***n*** | ***A*** | ***NE (total)*** | ***NE (mean)*** | ***NE 1*** | ***NE 2*** | ***NE ≥3*** | ***CT 1*** | ***CT ≥2*** | ***NE+ CT*** | ***M*** | ***1*** | ***2*** | ***3*** | ***4*** | ***≥5*** | ***n*** | ***Age (mean)*** | ***Comorbidity*** | ***n*** | ***Age (mean)*** | ***Comorbidity*** | ***NE+ CT*** | ***mean*** | ***0*** | ***1*** | ***2*** | ***≥3*** | ***mean*** |
| **Hospital type** | *General* | 2,916 (12) | 5,443 (22) | 7,826 (32) | 6,829 (28) | 1,767 (7) | 19,020,335  (41) | 1,044 (4) | 14,798 (60) | 1.8 | 8,621 (58) | 3,719 (25) | 2,458 (16) | 12,292 (49) | 587 (2) | 7,976 (32) | 1.6 | 4.292 (17) | 9,998 (40) | 5,061 (20) | 2,502 (10) | 2,928 (12) | 20,692 | 53 | 6,110 (30) | 4,042 (16) | 50 | 1,043 (26) | 2,018 (50) | 2.2 | 3043 (75) | 12 (0.3) | 920 (23) | 67 (2) | 1.8 |
|  | *Teaching* | 3,066 (12) | 5,888 (23) | 8,048 (32) | 6,717 (27) | 1,599 (6) | 7,282 (29) | 1,348 (5) | 14,607 (60) | 1.7 | 8,847 (61) | 3,724 (25) | 2,036 (14) | 12,351 (49) | 739 (3) | 7,828 (31) | 1.6 | 4149 (16) | 10,133 (40) | 5,096 (20) | 2,624 (10) | 3,316 (13) | 20,894 | 52 | 6,022 (29) | 4,369 (17) | 50 | 1,239 (28) | 2,161 (49) | 2.2 | 2572 (59) | 24 (0.5) | 1,659 (38) | 114 (3) | 1.7 |
|  | *Academic* | 571 (13) | 1,008 (23) | 1,428 (33) | 1,178 (27) | 191 (4) | 1,728 (39) | 278 (6) | 3,272 (75) | 2.3 | 1,411 (43) | 905 (28) | 956 (29) | 1,195 (27) | 54 (1) | 1,017 (23) | 1.9 | 707 (16) | 1,518 (35) | 899 (21) | 505 (12) | 747 (17) | 3,729 | 51 | 1,492 (40) | 630 (14) | 50 | 231 (37) | 306 (49) | 3.3 | 2,057 (41)‡ | <10 (<1) | 227 (36) | 146 (23) | 2.3 |
|  | *Private* | 346 (11) | 759 (25) | 999 (33) | 763 (25) | 185 (6) | 676 (22) | 91 (3) | 1,982 (65) | 1.6 | 1,314 (66) | 433 (22) | 235 (12) | 1,492 (49) | 62 (2) | 1,045 (34) | 1.5 | 731 (24) | 1,175 (39) | 616 (20) | 257 (8) | 273 (9) | 2,667 | 52 | 605 (23) | 377 (12) | 46 | 70 (19) | 239 (63) | 1.4 | 258 (69) | 119 (32) | <10 (<1) | <10 (<1) | 1.5 |
| **Region** | *West* | 2,137 (12) | 3,803 (22) | 5,550 (32) | 4,787 (27) | 1,188 (7) | 6,996 (28) | 1,190 (5) | 14,055 (57) | 1.66 | 8,661 (62) | 3,295 (23) | 2,099 (15) | 11,788 (48) | 578 (2) | 7,279 (29) | 1.6 | 4,186 (20) | 9,758 (39) | 5,147 (21) | 2,494 (10) | 3,221 (13) | 21,053 | 52 | 5,961 (21) | 3,692 (15) | 50 | 1,015 (27) | 1,812 (50) | 2.3 | 2,841 (77) | 134 (4) | 614 (17) | 103 (3) | 1.7 |
|  | *South* | 2,866 (12) | 5,862 (24) | 7,943 (32) | 6,549 (26) | 1,586 (6) | 4,249 (29) | 609 (4) | 9,089 (61) | 1.65 | 5,546 (61) | 2,227 (25) | 1,316 (14) | 7,664 (52) | 522 (4) | 5,176 (35) | 1.6 | 2,422 (20) | 5,946 (40) | 3,061 (21) | 1,555 (11) | 1,830 (12) | 12,138  12,138  12,138  12.138  12.138 | 52 | 3,511 (29) | 2,650 (18) | 49 | 727 (27) | 1,538 (58) | 2.2 | 1,404 (53)‡ | <10 (<1) | 1,132 (43) | 114 (4) | 1.7 |
|  | *North/East* | 1,850 (12) | 3,325 (22) | 4,657 (31) | 4,049 (27) | 933 (6) | 5,478 (31) | 961 (6) | 11,314 (65) | 1.97 | 5,799 (51) | 3,189 (28) | 2,326 (21) | 7,716 (44) | 411 (2) | 5,397 (31) | 1.7 | 3,094 (22) | 6,903 (40) | 3,386 (19) | 1,824 (10) | 2,258 (13) | 14,373 14.373 | 52 | 4,625 (32) | 3,054 (17) | 50 | 835 (27) | 1,415 (46) | 2.3 | 1,862 (61) | 18 (0.6) | 1,059 (35) | 115 (3) | 2.0 |

**Legend**: n: number of patients. A: allergy testing (skin prick test). NE: nasal endoscopy. CT: computed tomography scan of paranasal sinuses. †: no differences in mean age for region or hospital, with the exception of allergy: patients in academic hospitals were older (46) than patient in other hospitals (42 years for general/teaching and 43 years for private clinics). For admission days, mean age was not provided. ‡: <1% of data missing; pp: per patient.

1. Recommendations on diagnosis and treatment for adults with CRS, extracted from the CBO 2010 guideline (n=28).

| Number | Recommendation† | Level of evidence*‡* | Grade of recommendation*‡* | Comparable to Vektis data |
| --- | --- | --- | --- | --- |
| 1. | In patients with suspected CRS, patient history should be carefully considered, including symptoms, duration and severity of the complaints. | C | 3 | - |
| 2. | In patients with nasal polyps and complaints of CTS, it is advised to perform nasal endoscopy when symptoms last longer than three months and are severe. | C | 3 | + |
| 3. | In patient with complaints of CRS and no signs of disease at nasal endoscopy, a CT scan should be considered when symptoms persist. | C | 3 | - |
| 4. | An olfactory test can be helpful in patients with CRS and nasal polyps. | *D* | 4 | - |
| 5. | Before starting antibiotic treatment in patients with CRS, a culture from the middle nasal passages can be considered, especially when a patient  has undergone surgery. It is possible to start with an empirical antibiotic that can be adjusted if necessary. | B | 2 | - |
| 6. | When surgery is considered in patients with CRS, a pre-operative CT-scan should be mad with the aim of visualizing the anatomy and assess risks. | D | 4 | - |
| 7. | When radiographic imaging is indicated in patients with CRS, CT-scanning is the method of choice. | *C* | 3 | + |
| 8. | When making a pre-operative CT-scan in patients with CRS, the lowest radiation exposure possible should be chosen. | C | 3 | - |
| 9. | In patients with CRS and anamnestic symptoms of allergy, further diagnosis with RAST or skin prick test is indicated, because allergic rhinitis may be a contributing factor. | C | 3 | +/- |
| 10. | NO measurement does not yet play a role in patients with CRS and nasal polyps, since there are no studies showing the added value of NO measurement for these conditions. | B/C | 3 | - |
| 11. | Since the gold standard is invasive and laborious, NO measurement can be used in diagnosis primary ciliary dyskinesia. | B/C | 3 | - |
| 12. | The work-up of patients with CRS should not consist of diagnosing gastro-esophageal reflux. | C | 3 | - |
| 13. | In all patients with CRS, anamnesis should target lower airway disease. | B | 2 | - |
| 14. | Uncomplicated CRS should not be treated with short-term antibiotics (<14 days), unless in case of an acute exacerbation, then it can be considered. | B/C | 3 | - |
| 15. | There is insufficient evidence to make a recommendation on long-term antibiotic treatment (3 months) as an alternative to FESS, in patients that do not respond to local corticosteroids. The possible effect does not outweigh the risk of antibiotic resistance. | B | 3 | - |
| 16. | Local corticosteroids are first line treatment in patients with CRS, with or without nasal polyps. | A2 | 1-2 | - |
| 17. | If there are no contra-indications, patients with CRS can be treated effectively with systemic steroids during 14 days. | A2 | 1-2 | - |
| 18. | Is there is doubt to whether the loss of smell is sensory or mechanical, a diagnostic course of systemic corticosteroids for 14 days can be applied. | B | 3 | - |
| 19. | Due to a lack of randomized, controlled studies showing a relationship between GER and CRS, there is currently no place for anti-reflux treatment in CRS. | *C* | 3 | - |
| 20. | In patients with CRS without nasal polyps, it is desirable to start rinsing with isotonic saline. | A2 | 1 | - |
| 21. | Treatment with antihistamines in patients with CRS and nasal polyps is only effective in patients with allergy | A2  **B** | 2 | - |
| 22. | There is no place for the use of decongestives in patients with CRS | A2 | 2 | - |
| 23. | There may be a place for a trial treatment with anti-leukotrienes in CRS patients who also have asthma | B | 2 | - |
| 24. | CRS should initially be treated with extensive medical treatment. Surgical treatment should be reserved for patients not who do not respond adequately to medical treatment. | B | 3 | + |
| 25. | If CRS is treated surgically, FESS is the treatment of choice, rather than conventional open surgery | C | 3 | + |
| 26. | If there is an indication for diagnostic culture in patients with CRS, endoscopic culture of the middle meatus is preferred instead of antral lavage. | A2 | 2 | +/- |
| 27. | Antral lavage is not recommended as a treatment in CRS | B | 2 | + |
| 28. | In patients with therapy resistant CRS and insufficient improvement on medical therapy, revision surgery can be considered. | C | 3 | - |

**Legend**: †: translated from Dutch. *‡:* for details on level of evidence and grade of recommendation, see guideline^4^. +: yes. -: no. +/-: partially.
